# Protein–polyelectrolyte complexation: effects of sterically repulsive groups, macromolecular architecture and hierarchical assembly†

**DOI:** 10.1039/d4sm01254b

**Published:** 2024-12-06

**Authors:** Raman Hlushko, Alexander Marin, Alexander K. Andrianov

**Affiliations:** a Institute for Bioscience and Biotechnology Research, University of Maryland Rockville MD 20850 USA aandrianov@umd.edu

## Abstract

Self-assembly of proteins and polyelectrolytes in aqueous solutions is a promising approach for the development of advanced biotherapeutics and engineering efficient biotechnological processes. Synthetic polyions containing sterically repulsive ethylene oxide moieties are especially attractive as protein modifying agents, as they can potentially induce a PEGylation-like stabilizing effect without the need for complex covalent binding reactions. In this study, we investigated the protein-binding properties of anionic polyelectrolytes based on an inorganic polyphosphazene backbone, with ethylene oxide groups incorporated into both grafted and linear macromolecular topologies. The study was conducted in aqueous solutions using isothermal titration calorimetry, dynamic light scattering, and cryogenic electron microscopy to analyze the samples in their vitrified state. Our findings revealed that the stability of the resulting protein–polyion complexes and the thermodynamic profiles of these interactions were influenced by the molecular architecture of the polyions. Furthermore, the formation of hierarchical assemblies of polyions, through ionic crosslinking into nanogels, rapidly reduced or eliminated the ability of the polyelectrolyte to bind proteins. The comprehensive analysis, combining thermodynamic, spectroscopy and direct visualization techniques, provides valuable insights into the multivalent charge–charge interactions that are critical for the development of successful non-covalent protein modification methods.

## Abstract 2

Self-assembly of proteins and polyelectrolytes in aqueous solutions is a promising approach for the development of advanced biotherapeutics and engineering efficient biotechnological processes.

## Graphical abstract

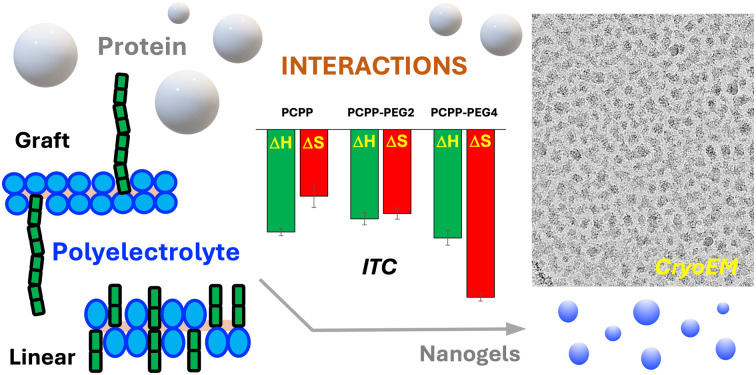

## Introduction

Modification of proteins through the formation of non-covalent assemblies with ionic polymers is a rapidly evolving approach at enhancing the performance of these biological macromolecules in life sciences applications.^1–8^ To achieve desirable characteristics, such as longer *in vivo* half-life, improved intracellular delivery or appropriate protein stability profile, the synthetic modifier must provide suitable biophysical functionality. To that end, polyions containing sterically repulsive ethylene oxide groups are of special interest, as their interactions with proteins may mimic the effects of PEGylation, which is a commercially successful technology that protects and stabilizes proteins by sterically shielding proteins with poly(ethylene glycol) (PEG) chains. This technology currently relies on covalent conjugation chemistry.^9–16^ The development of non-covalent approaches that leverage multivalent protein–polyion interactions^17–21^ presents an attractive alternative, as it can broaden the range of protein drug candidates while drastically reducing the high production costs associated with PEGylated drugs.^22^ The success of such an approach depends on meeting two critical conditions: first, the formation of complexes under the required conditions is feasible, and second, the dynamic stability of these complexes being adequate for the intended biomedical or biotechnological application.

One of the obvious challenges faced by researchers in the development of non-covalent protein modification using PEGylated polyions is overcoming the counteractive effect of sterically repulsive oligo(ethylene oxide) segments, which can impose spatial constraints on the attractive charge–charge interactions.^23,24^ This apparent intrinsic antagonism of the synthetic polymer modifiers can only be overcome by carefully optimizing the balance of functionalities and taking into account the differences between the production and application environments. Another factor that can play an important role in modulating protein–polymer interactions is the macromolecular architecture of the synthetic polyions, particularly the steric arrangement of inert or repulsive constituents.^17,18,25^ To this end, it is essential to investigate the effect of topological features, such as the graft *vs*. linear arrangement of protein-repulsive groups, number of junction nodes and the presence of hierarchical structures, on the dynamics of protein–polymer interactions. Indeed, studies on the complexation of linear and miktoarm star-shaped PEGylated polyelectrolytes with lysozyme already revealed that linear macromolecules were more efficient in the sequestration of lysozyme.^17^ Moreover, interactions of another PEGylated polyion with keratinocyte growth factor-2 were not dependent on the length of the grafted chains, but were suppressed for copolymers with higher graft density.^18^ However, due to the limitations of conventional polymerization processes, it remains challenging to obtain a side-by-side comparison of polymers with the same chemical composition, but varying macromolecular architecture.

**Chart 1:**
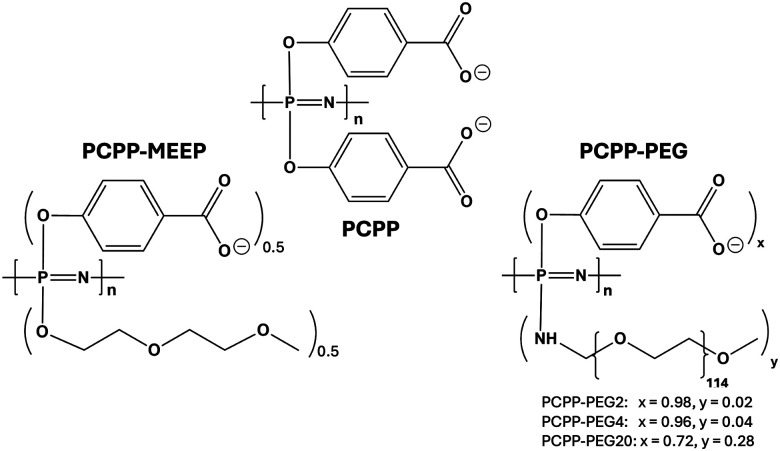
Chemical structures of PCPP (linear homopolymer), PCPP-MEEP (linear copolymer) and PCPP-PEG (graft copolymer).

Polyphosphazene chemistry is a synthetic pathway to achieve hybrid organo–inorganic polymers, which allows for a broad diversification of polymer structures *via* derivatization rather than polymerization routes, and offers an attractive platform for such studies.^26,27^ The suitability of polyphosphazene technology is further evidenced by the well-established ability of the water-soluble biodegradable polycarboxylates of this family to not only form complexes with proteins, but maintain their stability under physiological conditions.^7,28^ Poly[di(carboxylatophenoxy)phosphazene] (PCPP, Chart 1), in particular, has been extensively studied for its ability to spontaneously assemble with proteins in aqueous solutions with the formation of water-soluble complexes of nano-scale dimensions.^29–32^ Moreover, PCPP can be formed into nanoparticulate gels by its exposure to ionic cross-linkers, which can add an hierarchical dimension to a knowledge base of its interactions with proteins.^33–36^ A versatile synthetic platform allows for the insertion of ethylene oxide (EO) groups in the PCPP structure, either as graft PEG chains (PCPP-PEG) or as small dimers embedded in a linear polymer structure (PCPP-MEEP) (Chart 1).

The present paper reports on the effect of structural derivatization of polyphosphazene polyacid (PCPP) with sterically repulsive ethylene oxide moieties on its interactions with a model protein – hen egg lysozyme. The formation of protein–polymer complexes was studied using isothermal titration calorimetry (ITC) and dynamic light scattering (DLS) in aqueous solutions, as well as cryogenic electron microscopy (cryoEM) of samples in their vitrified state. The combined analysis of thermodynamics, spectroscopy and direct visualization data reveals the impact of the macromolecular topology on the strength of the intermolecular binding and dimensions of the resulting complexes, and on the mechanism of self-assembly. Furthermore, ionic cross-linking of a soluble PEGylated macromolecule into nanoscale-sized gel particles rapidly reduces or even entirely eliminates its ability to form complexes with a protein.

## Experimental

### Materials

Lysozyme from chicken egg white, BioUltra, lyophilized powder, ≥98% and bovine serum albumin, lyophilized powder, ≥96%, potassium chloride and sodium phosphate monobasic dihydrate (Sigma-Aldrich, Saint Louis, MO) and spermine tetrahydrochloride (Alfa Aesar, Ward Hill, MA) were used as received.

### Polymers

Polymers were synthesized as described previously (PCPP,^37^PCPP-MEEP^38^ and PCPP-PEG^39^) *via* ring opening polymerization of hexachlorocyclotriphosphazene and macromolecular substitution of the resulting polydichlorophosphazene. The polymer structure and composition were confirmed by ^1^H NMR and ^31^P NMR. Spectra were recorded using a 400 MHz Ascend Bruker NMR spectrometer equipped with a 400 MHz magnet (Bruker Biospin Corp, Billerica, MA).

Molecular weight (MW) analysis was performed using the asymmetric flow field flow fractionation (AF4) method. Characterization was carried out using a Postnova AF2000 MT instrument (Postnova Analytics GmbH, Landsberg, Germany). A regenerated cellulose membrane with a molecular mass cutoff of 10 kDa (Postnova, Germany) was used as a separation membrane, and 25 mM phosphate buffer (pH 7.4) was employed as the eluent. Molecular weights were determined using water-soluble polyphosphazene standards (17–1200 kDa range), which were obtained as described previously.^40^ Characterization results are shown in Table 1 and Fig. S1, ESI.†

**Table 1:** Polymer characterization data

Polymer	PEG (%, mol)	EO^a^ (%, mol)	EO^a^ (%, w/w)	MW^b^ (kDa)	Diameter^c^ (nm)	PDI^d^
PCPP	0	0	0	800	53	0.29
PCPP-PEG2	2	70	38	360	52	0.33
PCPP-PEG4	4	83	56	165	67	0.42
PCPP-PEG20	28	98	91	—e	12	0.40
PCPP-MEEP	—	71	39	600	40	0.27

a Ethylene oxide (EO) groups.

b Molecular weight as determined by AF4.

c *Z*-average hydrodynamic diameter as determined by DLS.

d Polydispersity index as determined by DLS.

e The molecular weight of PCPP-PEG20 was not determined due to strong non-specific interactions during the analysis.

### Isothermal titration calorimetry (ITC)

ITC experiments were performed using a Nano ITC SV instrument (TA Instruments, Waters, New Castle, DE, USA) in an aqueous solution (50 mM phosphate buffer, pH 7.5) at 25 °C. In a typical experiment, 0.125 mg mL^−1^ polymer solution was placed into an isothermal chamber, and was titrated by 10 μL aliquots of 2.5 mg mL^−1^ lysozyme from a 250 μL syringe rotating at 36.7 rad s^−1^ with a 300 s delay between each injection. Each injection generated a heat release curve (microjoules per second *versus* seconds), which later was processed using NanoAnalyze software, version 3.12.5 (TA Instruments, Waters, New Castle, DE, USA) to yield the heat associated with each injection. Data analysis was performed with the above software, and a single set of identical sites (SSIS) binding model was used to calculate the thermodynamic parameters: binding constant (*K*_d_), reaction stoichiometry (*n*), enthalpy (Δ*H*), and entropy (Δ*S*).

### Dynamic light scattering (DLS)

DLS studies of the polymer–protein interactions were conducted using a Malvern Zetasizer Nano ZS instrument. The data were recorded and analyzed using Malvern Zetasizer 7.10 software (Malvern Instruments Ltd, Worcestershire, U.K.). Samples were filtered using Millex 0.22 mm filters prior to analysis.

### Cryogenic electron microscopy (cryoEM)

An aliquot of aqueous polymer solution was deposited on holey carbon film TEM grids (Q3100CR1.3–2 nm, Electron Microscopy Sciences, Hatfield, PA), which were pre-treated with plasma using glow discharge PELCO EasiGlow (Ted Pella Inc., Redding, CA). The sample-containing grids were then double-blotted on the Vitrobot (Vitrobot Mark IV, FEI, Hillsboro, OR) and vitrified in liquid ethane. The visualization was conducted using 200 kV Talos Arctica (FEI, Hillsboro, OR) equipped with FEI Falcon3EC direct electron detector. Imaging was performed at a temperature of about 90 K at an acceleration voltage of 200 kV. The data were collected using EPU software and processed in CryoSPARC 4.2.1 (Structura Biotechnology Inc., Toronto, Canada).

## Results and discussion

### Effect of the steric arrangement of repulsive ethylene oxide groups – graft *vs*. linear topology

**Fig. 1:**
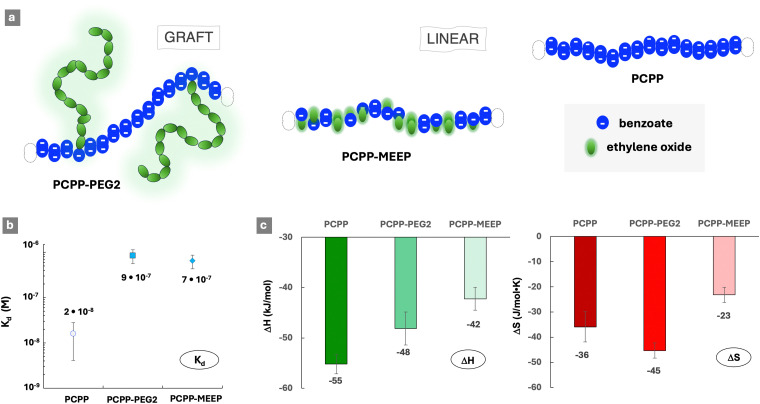
(a) Schematic of copolymers containing ethylene oxide and benzoate groups (graft PCPP-PEG2 and linear PCPP-MEEP) and homopolymer containing solely benzoate groups – PCPP. (b) Dissociation constants of lysozyme complexes with PCPP, PCPP-PEG2 and PCPP obtained by ITC, and (c) thermodynamic patterns of interactions between lysozyme complexes and polymers – PCPP, PCPP-PEG2 and PCPP-MEEP (Δ*H* – enthalpy change, Δ*S* – entropy change; favourable contributions are shown in green, unfavourable in red, ITC titration of polymers with the protein: 0.125 mg mL^−1^ polymer, 4.0 mg mL^−1^ protein, 50 mM phosphate buffer, pH 7.5).

The affinity of polyphosphazenes to lysozyme was achieved utilizing anionic benzoate side groups of PCPP (Chart 1) as protein binding moieties. Structural modification of the PCPP-based polyacids with sterically repulsive ethylene oxide moieties was realized by exploiting two distinctly different approaches (Chart 1 and Fig. 1a). First, the PCPP-PEG2 graft copolymer was synthesized *via* covalent attachment of oligomeric PEG chains (5 kDa) to a polyphosphazene backbone. Second, short methyl-capped ethylene oxide dimers were utilized as polymer side groups, thereby maintaining the linear topology of the resulting PCPP-MEEP copolymer. Both macromolecules were designed to contain approximately 70% (mol) of ethylene oxide groups, which corresponds to 2% (mol) of PEG graft chains in the PCPP-PEG copolymer (Table 1). The ability of these polymers to bind a model protein, lysozyme, was evaluated by isothermal titration calorimetry (ITC) and benchmarked against that of a linear PCPP homopolymer. The quantitative label-free ITC method allows for examination of both binding isotherms and thermodynamics of interactions, from which the nature of protein–polymer complexation can be explored.^1,41^

The ITC results demonstrate that, despite the relatively high content of sterically repulsive moieties, both copolymers were able to spontaneously self-assemble with lysozyme in aqueous solutions (Fig. S2–S4, ESI†). However, the dissociation constants (*K*_d_) of complexes formed by both copolymers (Fig. 1b) were over ten-fold higher than that of a PCPP-lysozyme system (2 × 10^−8^ M), which is in line with previous findings for that system.^29,32^ Somewhat surprisingly, only a minor difference was detected in the stability of complexes formed by graft and linear copolymers – with *K*_d_ values equal to 9 × 10^−7^ M and 7 × 10^−7^ M, correspondingly. This suggests that ethylene oxide moieties embedded into linear polymer chains can be as effective as graft PEG architectures in suppressing the interactions that are characteristic for the PCPP homopolymer (2 × 10^−8^ M).

Analysis of the thermodynamic patterns (Fig. 1c) reveals a noticeable distinction in the mechanism of protein complexation displayed by the two topologically different polymers. As seen from the figure, the protein complexation in all studied systems was characterized by the compensation of an enthalpic gain, which is a driving force for the process, through an unfavorable change in the entropy. The observed thermodynamic profile is consistent with those generally reported for protein–polymer assembly processes driven by electrostatic forces.^1,42^ In interactions with the cationic lysozyme, PCPP predictably demonstrated the highest enthalpy gain compared to both copolymers, and the PCPP-MEEP interactions were characterized by the least favorable change. The above results can be interpreted by considering the differences in the linear charge distribution in these polymers. A homopolymer (PCPP) presents an uninterrupted sequence of carboxylic acid groups (Fig. 1a). The PCPP-PEG2 copolymer retains lengthy blocks of charges that are interrupted by only 2% (mol) of 5 kDa PEG grafts, which are responsible for the 70% (mol) content of ethylene oxide groups in this copolymer (Table 1). In contrast with PCPP-PEG2, the same 70% (mol) of ethylene oxide groups in the linear PCPP-MEEP copolymer are randomly distributed along the backbone, causing a dramatic reduction in the charge density and its distribution (Fig. 1a and Table S1, ESI†). Therefore, it can be concluded that the higher density and blocky distribution patterns of the ionic groups in the polymers promote stronger electrostatic protein–polymer interactions. This is in line with the previously reported phenomena of cooperativity and associative charging,^43,44^ as well as the effects of charge density^8^ resulting in most favorable energetic contributions to self-assembly processes.

The comparison of entropic losses for the PCPP-PEG and PCPP-MEEP interactions with lysozyme reveals that the enthalpic benefits resulting from the blocky distribution of ionic groups in a graft copolymer are accompanied with entropic penalties (Fig. 1c). Although entropic contributions are generally less important than energetic ones for interactions involving weak polyelectrolytes,^44^ changes in the entropy, which are usually associated with the release of counterions from the vicinity of macromolecules^41,45^ should not be overlooked. The lower charge density of PCPP-MEEP, which is already noted above for the less favorable energetic contribution, is expected to weaken the counterion–polymer binding.^44^ Therefore, the linear copolymer is more susceptible to the release of counterions. This provides some compensation to the overall unfavorable entropy contribution in the case of PCPP-MEEP, which is seen in Fig. 1c.

**Fig. 2:**
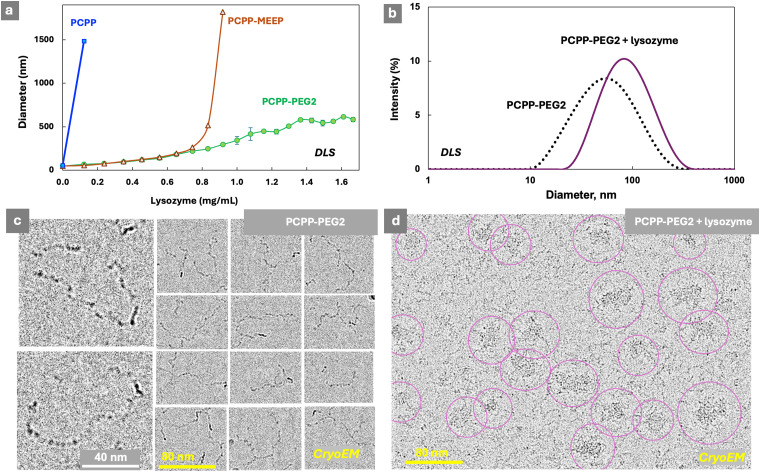
(a) Hydrodynamic diameter of polymers upon titration with lysozyme (DLS, *z*-average diameters shown, 0.5 mg mL^−1^ polymer, 50 mM phosphate buffer), (b) DLS profiles of PCPP-PEG2 and its mixture with lysozyme, and cryoEM images of (c) PCPP-PEG2 and (d) PCPP-PEG2 – lysozyme (0.25 mg mL^−1^ polymer, 0.55 mg mL^−1^ protein, 50 mM phosphate buffer, pH 7.5).

To complement the analysis of interactions in such systems, solution characteristics and morphology were also reviewed. Dynamic light scattering (DLS) studies reveal that the titration of PCPP and PCPP-MEEP with lysozyme is characterized by a sharp phase transition (Fig. 2a). In contrast, the protein self-assembly with PCPP-PEG2 may be described as a slow increase in the size of agglomerates, which still maintain a relatively narrow unimodal distribution (Fig. 2a and b). CryoEM images of PCPP-PEG2 and its complexes with lysozyme in their vitrified state show a transition from a coil-like chain of the polymer to micelles (Fig. 2c and d and Fig. S5, ESI†). This phenomenon was not observed for PCPP and PCPP-MEEP systems, and should be attributed to the stabilizing effect of graft PEG chains.

### Effect of the PEG graft density

**Fig. 3:**
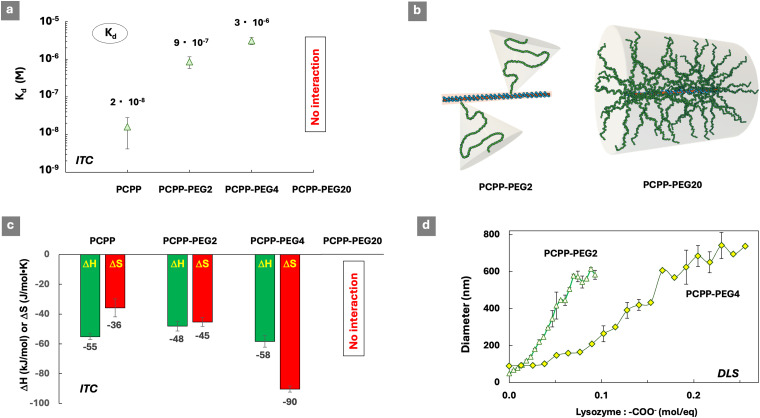
(a) Effect of the PEG graft density on the dissociation constants of lysozyme complexes with PCPP-PEG copolymers and PCPP. (b) Schematic showing a multi-fold increase in the PEG density in PCPP-PEG copolymers. (c) Thermodynamic signatures of protein–polymer interactions (DH – enthalpy change, DS – entropy change; favorable contributions are shown in green, unfavorable in red, ITC titration of polymers with the protein, 0.125 mg mL^−1^ polymer, 4.0 mg mL^−1^ protein, 50 mM phosphate buffer, pH 7.5) and (d) hydrodynamic diameter of polymers upon titration with lysozyme (DLS, *z*-average diameters shown, 0.5 mg mL^−1^ polymer, 50 mM phosphate buffer).

The effect of varying the graft density was studied using PCPP-PEG copolymers containing 2%, 4% and 20% (mol) of PEG chains (Table 1). Similar to PCPP-PEG2, the PEGylated copolymer contains 4% (mol) of PEG grafts, and was capable of binding lysozyme in aqueous solution (Fig. S6, ESI†). The complex dissociation constants determined by ITC (Fig. 3a) increase in the following series: PCPP > PCPP-PEG2 > PCPP-PEG4, which is in line with the anticipated growth in a steric barrier due to the increasing density of the PEG chains. No protein–polymer interactions were observed by ITC for high graft density PCPP-PEG20. A schematic shown in Fig. 3b outlines the multi-fold increase in the PEG density in PCPP-PEG copolymers, and provides an illustration for the potential steric challenges faced by protein molecules in accessing the anionic side groups located near the backbone of the highly grafted copolymer. However, the compact structure of PCPP-PEG20 revealed by DLS and its unusual behavior in AF4 analysis (Table 1) obscure further analysis of the interactions in this system.

The thermodynamic patterns of interactions (Fig. 3c) reveal a progressive increase in the unfavorable losses in entropy when the graft density increases from 0% to 4% (mol). Since entropic changes in such systems are typically attributed to the release of counterions, it is reasonable to assume that the higher graft density promotes the association of small ions with the polymer. This may be explained by their retention through the well-known phenomenon of association of metal ions with ethylene oxide-containing molecules.^46^ Accordingly, no entropy gain can be expected, as the released counterions still remain associated with the polymer, although henceforth through their complex formation with PEG grafts. Another potential explanation can stem from the experimentally observed smaller dimensions of protein–polymer complexes in the PCPP-PEG4 system under the same overall conditions (Fig. 3d). These morphological differences in the system can potentially lead to a smaller ionic core size in PCPP-PEG4 complexes, leading to higher spatial charge density and stronger retention of counterions.

The analysis of the protein-binding properties of PCPP-PEG systems cannot be completed without reviewing the potential effect of intramolecular bond formation in these graft copolymers. This prerequisite stems from the previously observed ability of PCPP to form hydrogen bonded interpolymer complexes with poly(ethylene oxide) at near physiological conditions.^47^ To that end, interactions between PCPP and 5 kDa PEG, which was used in the synthesis of PCPP-PEG copolymers, were studied by ITC. Although the formation of the complex was detected (Fig. S7, ESI†), the dissociation constant (4 × 10^−4^ M) was found to be orders of magnitude higher than those observed for lysozyme–polymer complexes (Fig. 1b and 3a). Furthermore, the presence of unbound PEG in the PCPP – lysozyme titration system did not have a noticeable effect on the dissociation constant of the protein–polymer complex (Fig. S8, ESI†). It is noteworthy that the entropy loss in the PCPP-PEG mixture titrated by lysozyme was minimized compared to PCPP in the absence of PEG (Fig. S8c, ESI†). This can indicate the release of PEG from a PCPP-PEG complex in a PEG-lysozyme ligand exchange reaction. The results confirm that the intermolecular interactions in PEGylated polyions cannot be neglected. However, the increase in entropy losses with increasing PEG graft density observed above (Fig. 3c) is not associated with such phenomenon.

### Ionotropic nanogels of graft copolymers and their interactions

**Fig. 4:**
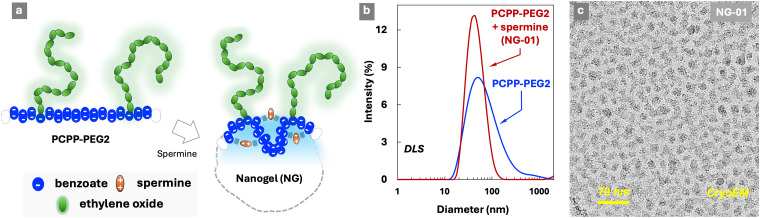
(a) Schematic of the formation of spermine cross-linked PCPP-PEG2 nanogels – NGs, (b) DLS profiles of PCPP-PEG2 and PCPP-PEG2 crosslinked with spermine – NG (1 mg mL^−1^PCPP-PEG2, 1 mg mL^−1^ spermine, 50 mM phosphate buffer, pH 7.4) and (c) cryoEM images of PCPP-PEG2 cross-linked with spermine (1 mg mL^−1^PCPP-PEG2, 1 mg mL^−1^ spermine, 50 mM phosphate buffer, pH 7.4).

Ionic cross-linking of polyelectrolytes is a well-established approach to engineer hierarchical (three-dimensional) supramolecular structures, which find utility in a variety of life sciences and industrial applications.^48–51^ To that end, ionic polyphosphazenes present an attractive opportunity due to their ability to form hydrogels by undergoing cross-linking in the presence of physiologically benign multivalent cation – spermine.^33,52^ The resulting assemblies can be formed into nanoparticles of various architectures for use in the areas of drug delivery and theragnostics.^34–36^ However, the effect of such hierarchical assembly on the ability of the polymer to form complexes with proteins has not yet been elucidated. Ionotropic gelation of PCPP-PEG2 in the presence of spermine is manifested in some changes in DLS profiles and the formation of nano-sized gels, which are clearly seen in the images of vitrified samples (Fig. 4 and Fig. S9, ESI†). The cryoEM method is especially effective in illustrating a striking transition from the coil-like chains of PCPP-PEG (Fig. 2c) to the discrete and compact assemblies formed by this polymer upon addition of spermine (Fig. 4c and Fig. S9, ESI†).

**Fig. 5:**
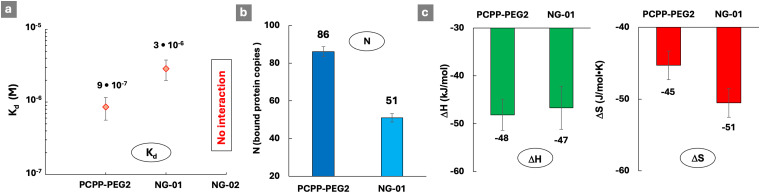
ITC titration of spermine cross-linked PCPP-PEG2 nanogels with lysozyme: (a) dissociation constants, (b) number of protein molecules bound to a single PCPP-PEG2 chain and (c) thermodynamic patterns of interactions (0.125 mg mL^−1^ nanogel; 2.5 mg mL^−1^ protein; NG-01 and NG02: 1 and 2 mg mL^−1^ spermine, correspondingly; 50 mM phosphate buffer; pH 7.5).

The ability of such sterically constraint nanostructures to interact with lysozyme was investigated by ITC at two concentrations of the crosslinker (0.1% and 0.2% spermine – nanogels NG-01 and NG-02, correspondingly), and was benchmarked against soluble PCPP-PEG2. An approximately three-fold increase in the dissociation constant of the complex with the protein was detected for a lightly cross-linked NG-01 nanogel when compared to a water-soluble polymer (Fig. 5a and Fig. S10, ESI†). Furthermore, a complete loss of the protein-binding ability was uncovered when the cross-linking density was increased by only two-fold. The assessment of a number of available protein-binding sites (*N*), when calculated per a single chain of PCPP-PEG2, shows that the spatial limitations caused by light crosslinking resulted in their approximately 40% decrease (Fig. 5b). Thermodynamic profiles of the interactions reveal (Fig. 5c) that the enthalpy contribution remains practically the same for both concentrations of spermine, which suggests that the observed effect is not associated with weakened ionic interactions due to a change in the overall polymer charge introduced by a cross-linker. Instead, the main impact of cross-linking was associated with the less favorable entropy change observed for nanogels (Fig. 5c). This may be explained by a more compact matrix of the crosslinked system, resulting in a higher density of negative charges and enhanced retention of counterions. It is noteworthy that substantial changes in both reactivity and structural characteristics of the cross-linked polymer revealed by ITC and cryoEM methods manifested only in subtle variations detected by DLS.

### Ion effects on interactions of a PEGylated polymer

**Fig. 6:**
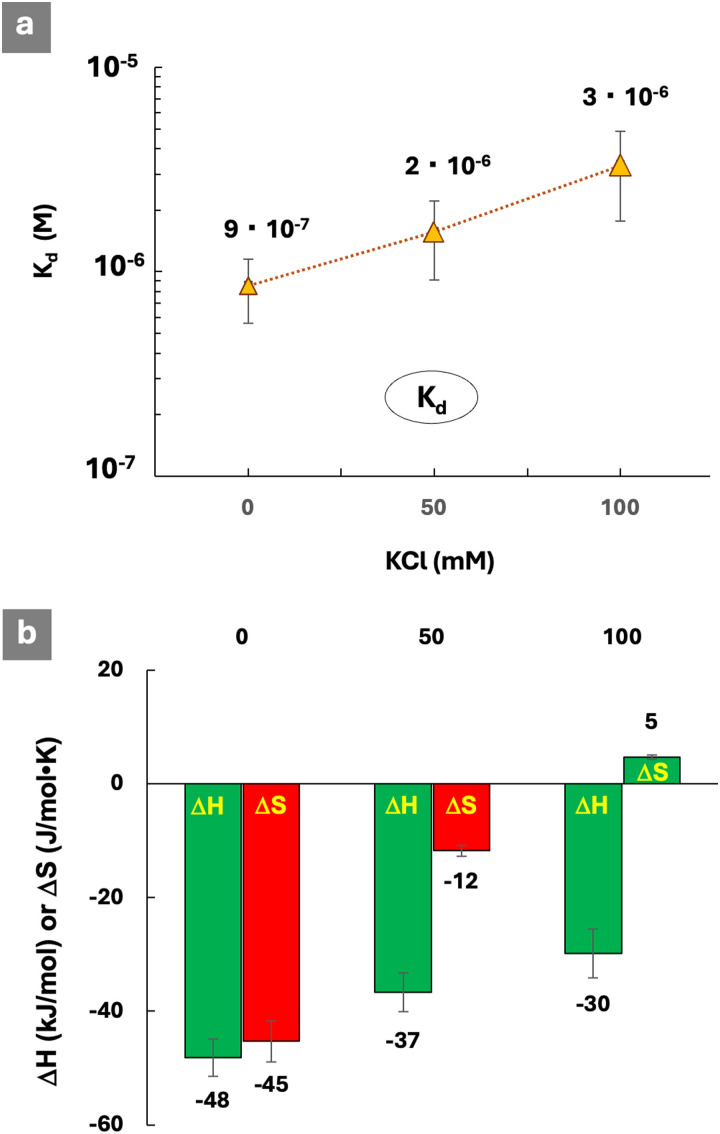
ITC titration of PCPP-PEG2 with lysozyme in the presence of added potassium chloride: (a) dissociation constants and (b) thermodynamic patterns of interactions (0.125 mg mL^−1^PCPP-PEG2; 2.5 mg mL^−1^ protein; 50 mM phosphate buffer; pH 7.5).

As discussed above, many observations and trends made on the structure–protein binding activity relationship were associated with changes in entropy, and attributed to the specifics of counterion release-retention mechanisms in the system. To confirm the validity of such assumptions, we explored the effect of ionic strength on the stability of lysozyme-PCPP-PEG2 complexes and thermodynamic patterns of interactions in the presence of various concentrations of potassium chloride (Fig. 6 and Fig. S11 and S12, ESI†).

As seen from Fig. 6a, the dissociation constants gradually increase when the salt concentration increases in the 0–100 mM range. The decrease in the beneficial change in enthalpy is observed at higher concentrations of salt (Fig. 6b). This is expected for reactions driven by electrostatic interactions, which are progressively screened at higher concentrations of potassium chloride. No protein–polymer interactions are observed when the concentration of the added salt reaches 500 mM. The most dramatic effect is observed for entropy changes, which is reversed from unfavorable (no added potassium chloride) to favorable at 100 mM of salt (Fig. 6b). This is in line with the above suggested assumptions and literature data, indicating that entropy changes are driven mainly by a counterion release that is facilitated by the higher ionic strength.^1,41,53,54^ The high sensitivity of the system to the effects of the ions also emphasizes the need to undertake the evaluation of protein–polymer interactions at the ionic strength dictated by the needs of the specific application.

## Conclusions

Structurally diverse polyions engineered on the basis of an inorganic phosphorus and nitrogen skeleton offer a convenient tool for investigating the effect of molecular topology on protein–polymer interactions. A side-by-side comparison of the protein binding by graft (PCPP-PEG) and linear (PCPP-MEEP) copolymers with identical chemical composition is a representative example of the insights that polyphosphazenes can potentially provide. Somewhat unexpectedly, in our study performed by an ITC method, polyanions with either graft or main chain localization of sterically repulsive ethylene oxide groups formed complexes, which showed comparable stability. Despite similar dissociation constants of such protein–polymer assemblies, the thermodynamic patterns of the interactions displayed significant differences. In both cases, the compensation of an enthalpic gain by an unfavorable change in the entropy was observed. In contrast with the graft copolymer, the interactions of a linear macromolecule were characterized by a smaller energy contribution. However, this was favorably offset by a significantly lower entropy loss. Mechanistically, these topology-driven changes can be associated with a shifting balance between the contributions of electrostatic interactions of macromolecular partners and counterion expulsion in the system. In future studies, it may be interesting to investigate whether the observed mechanistic differences can have an impact on the stability of complexes in solutions of various ionic strength, and especially, in the complex protein-rich physiological environment.

Another practically important result is in the demonstrated rapid drop in the polymer protein-binding capacity upon its ionic cross-linking and transformation to a nanogel. Further studies can shed light on whether this apparently disappointing outcome can be turned to an advantage by the gelation of the already-formed protein–polymer complex. It can be envisioned that such approach will lead to a superior stability of complexes in a protein-rich environment, in which undesirable competitive exchange reactions are expected. Lastly, the importance of the multi-method approach for studying protein–polymer interactions needs to be emphasized. It is evident that a combination of ITC and cryoEM techniques in the analysis of the cross-linked system was critical in providing information, on which the commonly used DLS characterization was essentially silent.

Overall, multifunctional polyions containing sterically repulsive ethylene oxide moieties remain some of the most attractive choices for the non-covalent modification of proteins. They have been intensively researched in the areas of protein stabilization, purification, cellular uptake and long-circulating stealth biotherapeutics. The technology can potentially offer desirable dynamic protection of the protein, simple “mix-and-match” formulation approaches, reduction in manufacturing costs and streamlining regulatory processes. However, the pathway toward a successful commercial development is critically dependent on the researcher's ability to effectively control the stability of the resulting assemblies in the environment dictated by a specific biomedical or biotechnological application.

## Author contributions

Conceptualization: A. K. A.; investigation: R. H. and A. M., data curation, formal analysis R. H.; writing – original draft preparation, writing – review and editing, supervision, project administration, funding acquisition: A. K. A.

## Data availability

The data supporting this article have been included as part of the ESI.†

## Conflicts of interest

There are no conflicts to declare.

## Supplementary Material

SM-021-D4SM01254B-s001
