# Correction: Protein–polyelectrolyte complexation: effects of sterically repulsive groups, macromolecular architecture and hierarchical assembly

**DOI:** 10.1039/d5sm90071a

**Published:** 2025-04-24

**Authors:** Raman Hlushko, Alexander Marin, Alexander K. Andrianov

**Affiliations:** a Institute for Bioscience and Biotechnology Research, University of Maryland Rockville MD 20850 USA aandrianov@umd.edu

## Abstract

Correction for ‘Protein–polyelectrolyte complexation: effects of sterically repulsive groups, macromolecular architecture and hierarchical assembly’ by Raman Hlushko *et al.*, *Soft Matter*, 2025, **21**, 418–426, https://doi.org/10.1039/D4SM01254B.

The authors regret the omission of a funding acknowledgement in the original article. This acknowledgement is given below.

Alexander K. Andrianov would like to acknowledge financial support from the NIH under the Grant R01 AI168048.

The Royal Society of Chemistry apologises for these errors and any consequent inconvenience to authors and readers.

