# Supplementary material for: Protein–polyelectrolyte complexation: effects of sterically repulsive groups, macromolecular architecture and hierarchical assembly
Source: Soft Matter. 2024 Dec 6;21(3):418–26. doi: 10.1039/d4sm01254b (PMC12005243; doi:10.1039/d4sm01254b)
Supplement: SM-021-D4SM01254B-s001 [file SM-021-D4SM01254B-s001.pdf]

Supplementary Information (SI) for Soft Matter.
This journal is © The Royal Society of Chemistry 2024

Protein-Polyelectrolyte Complexation – Effects of Sterically Repulsive Groups, Macromolecular Architecture and Hierarchical Assembly

Raman Hlushko, Alexander Marin and Alexander K. Andrianov

Institute for Bioscience and Biotechnology Research, University of Maryland, Rockville, MD 20850 USA

Supplementary Information

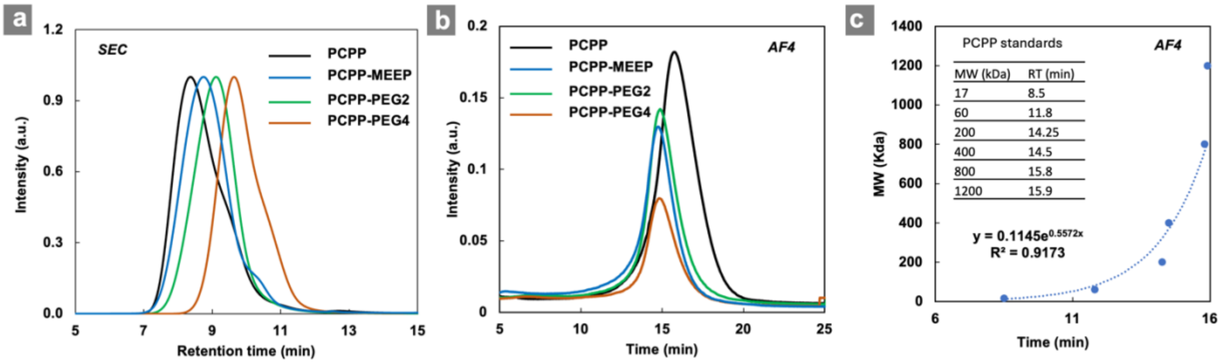

Fig. S1. (a) DLS profiles, (b) AF4 fractograms of PCPP, PCPP-MEEP, PCPP-PEG2 and PCPP-PEG4 and (c) AF4 calibration curves using PCPP standards (0.5 mg/mL polymer, 50 mmol phosphate buffer, pH 7.4).

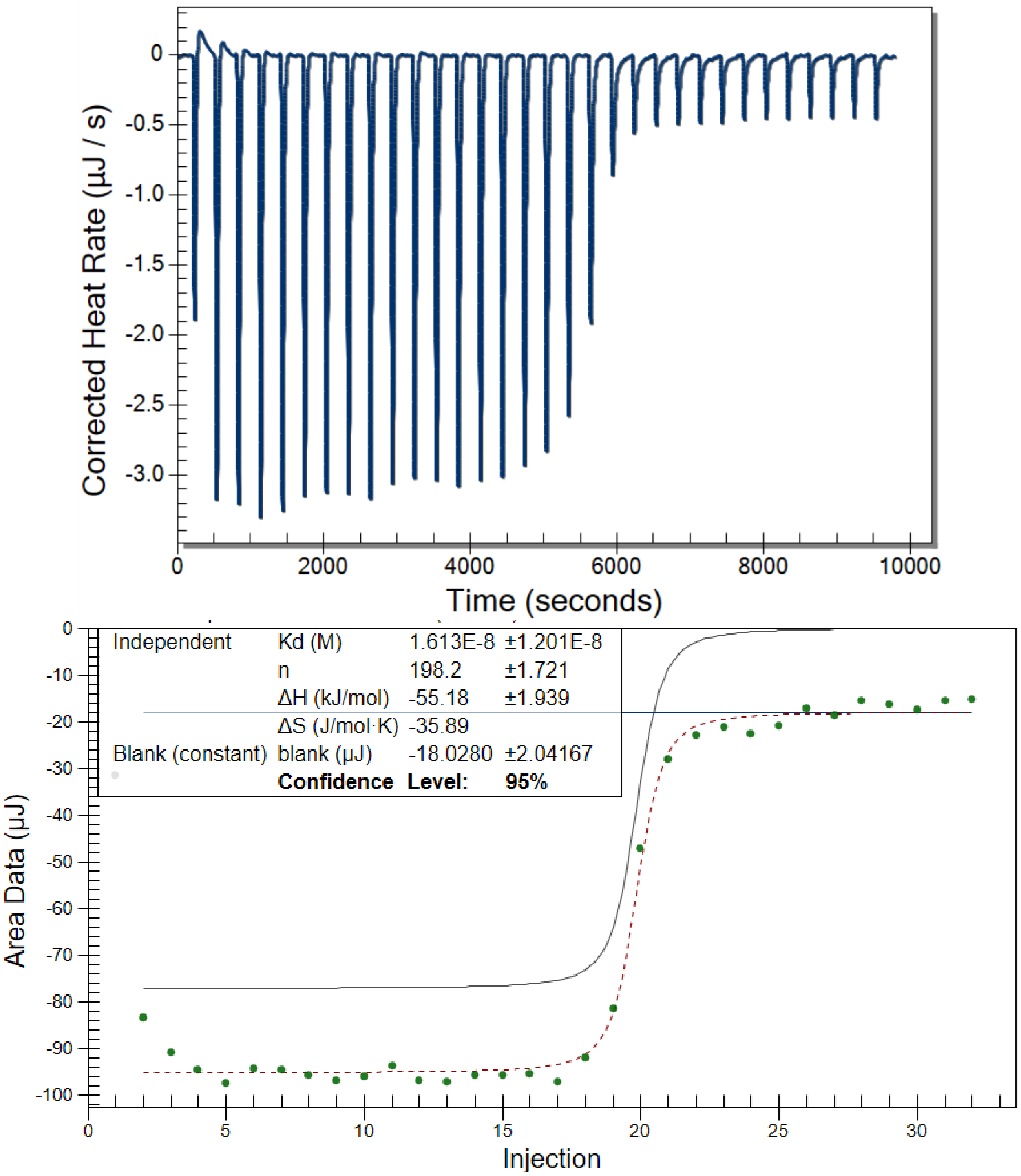

Fig. S2. ITC titration of PCPP with lysozyme (0.125 mg/mL polymer, 2.5 mg/mL protein, 50 mM phosphate buffer, pH 7.5)

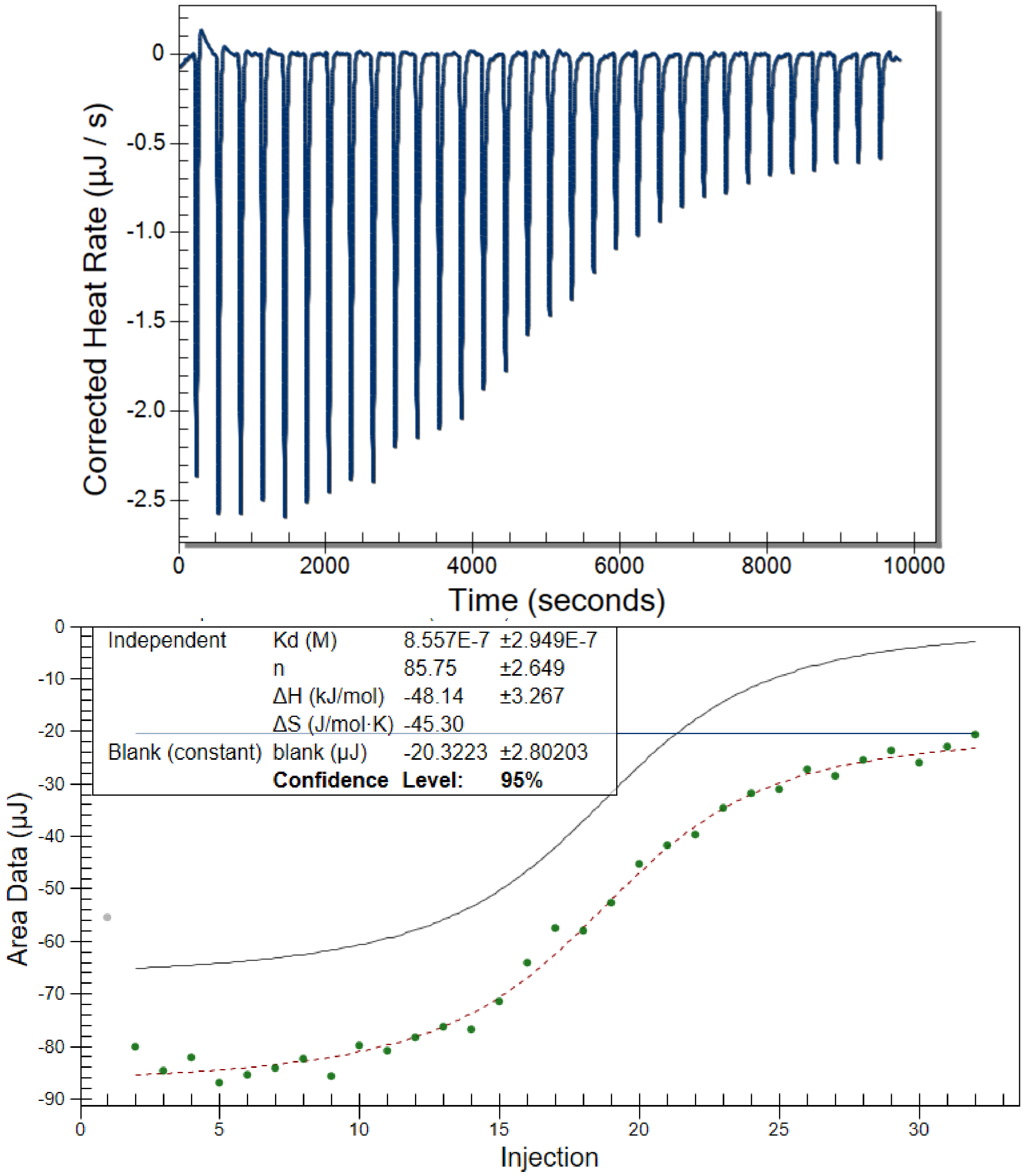

Fig. S3. ITC titration of PCPP-PEG2 with lysozyme (0.125 mg/mL polymer, 2.5 mg/mL protein, 50 mM phosphate buffer, pH 7.5)

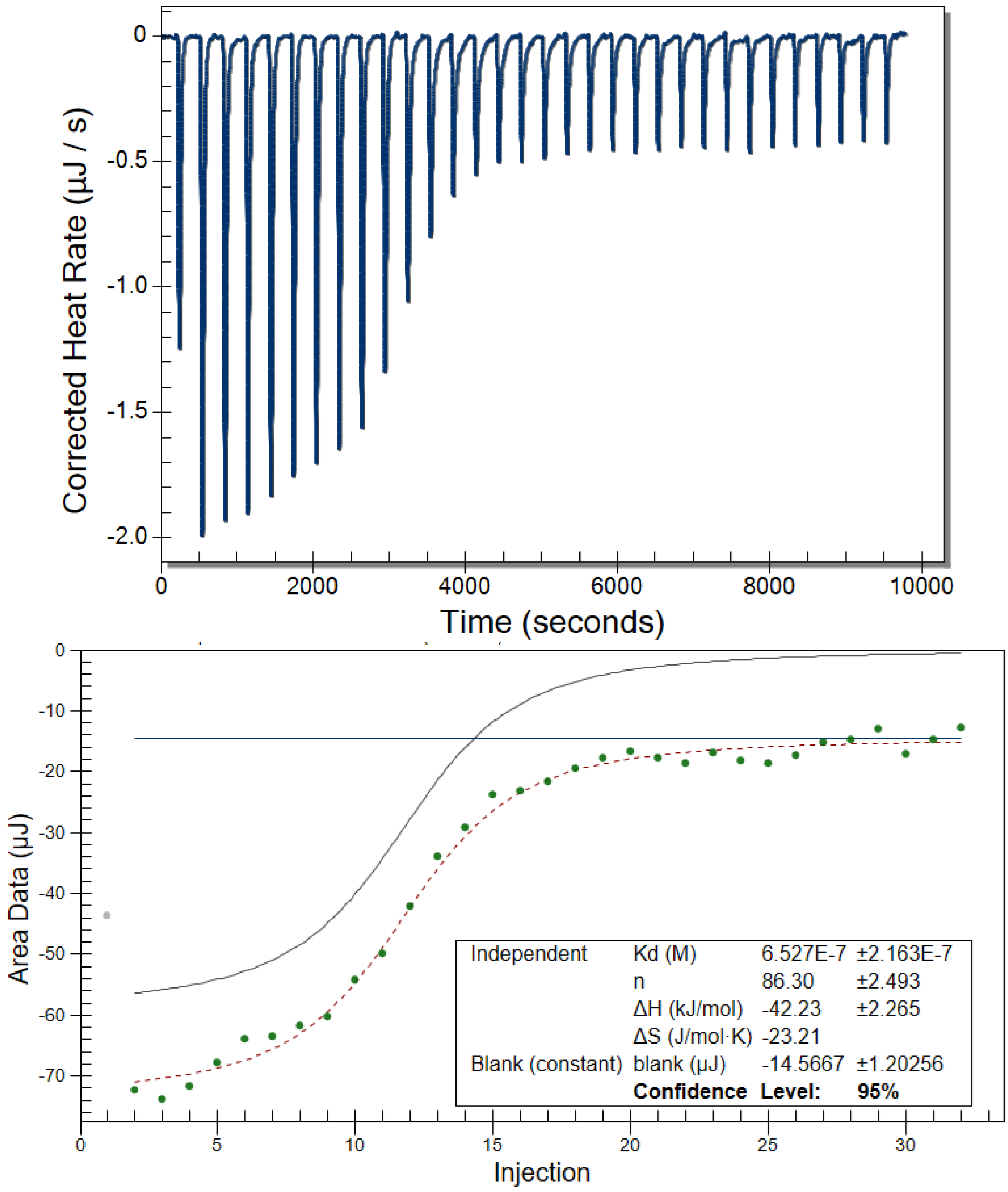

Fig. S4. ITC titration of PCPP-MEEP with lysozyme (0.125 mg/mL polymer, 2.5 mg/mL protein, 50 mM phosphate buffer, pH 7.5)

Table S1. Assessment of apparent distances between adjacent charges in PCPP-PEG2 and PCPP-MEEP copolymers

Polymer	MW[a] (kDa)	mw_r[b]	N[c]	e[d]	L[e] (Å)	b[f] (Å)
PCPP-PEG2	360	521	691	1353	2182	1.6
PCPP-MEEP	600	305	1967	1967	6216	3.2

[a] Molecular weight of the polymer; [b] molecular weight of the polymer repeat unit; [c] degree of polymerization; [d] number of elementary charges (carboxylate ions) per polymer chain; [e] polymer counter chain length - calculated assuming 1.58 as the average length of polyphosphazene skeleton bond (H. R. Allcock, *Chemistry and Applications of Polyphosphazenes*, Wiley, Hoboken, NJ, 2002, page 405); [f] apparent distance between adjacent charges in the polymer chain (b=L/e).

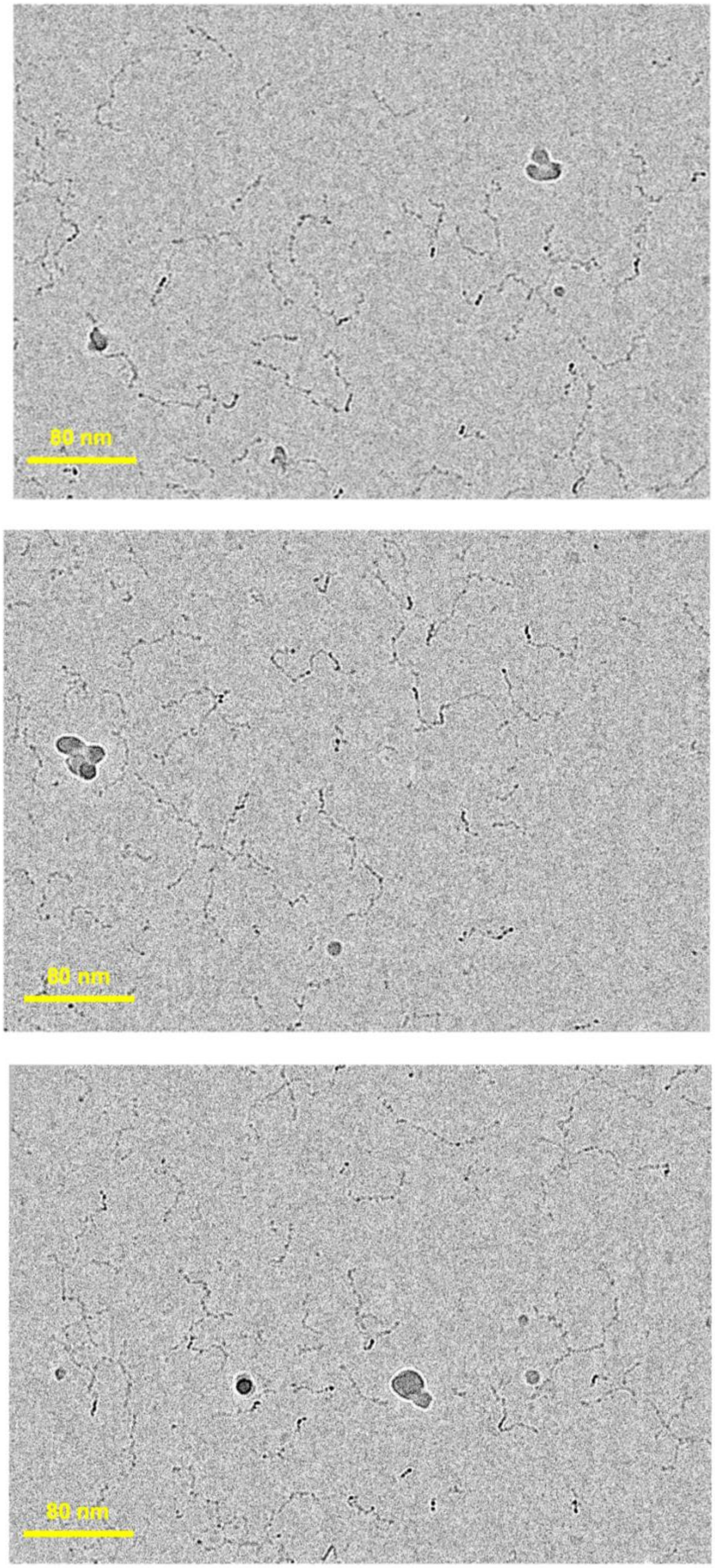

Fig. S5. cryoEM images of PCPP-PEG2 (0.25 mg/mL polymer, 50 mM phosphate buffer).

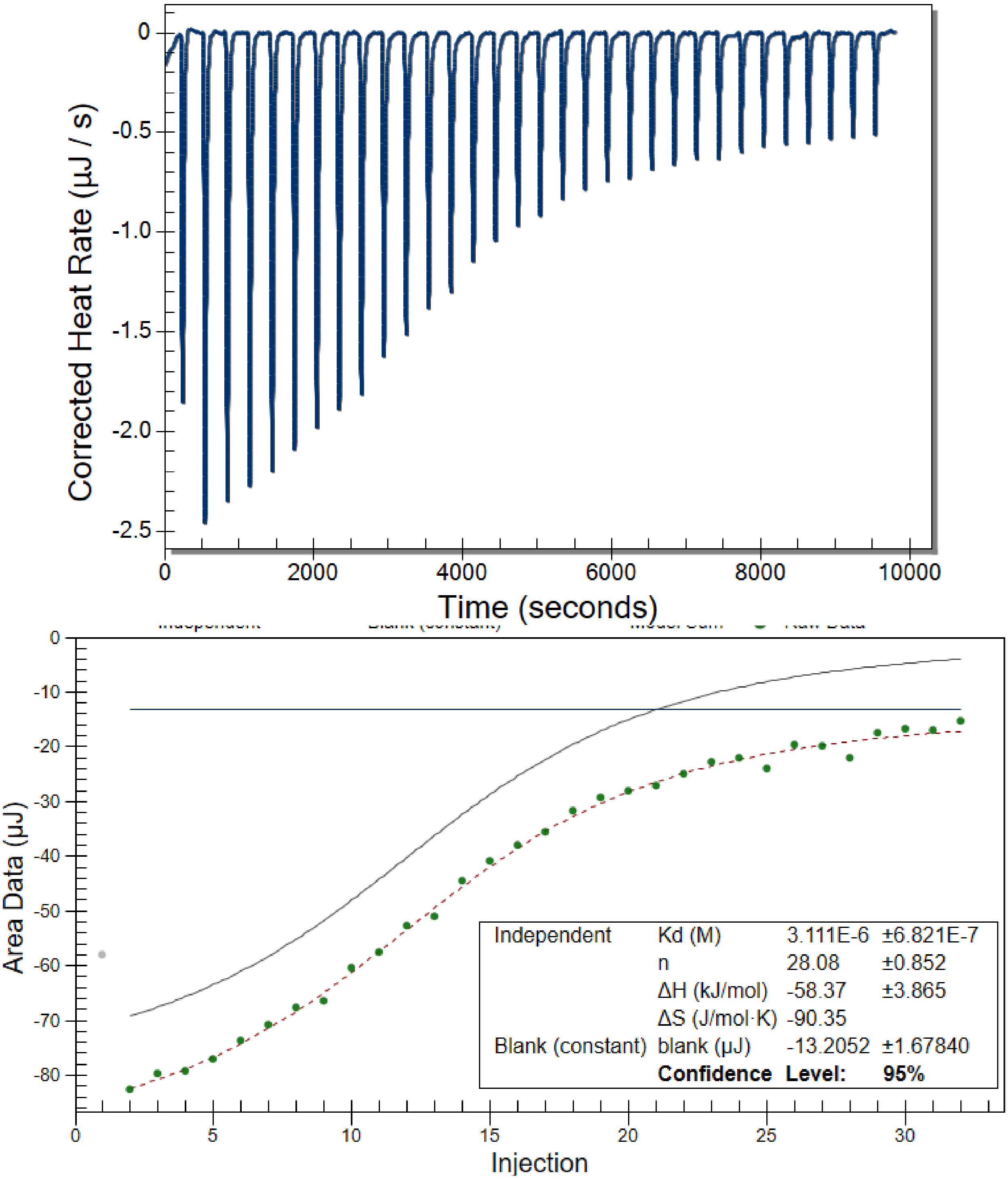

Fig. S6. ITC titration of PCPP-PEG4 with lysozyme (0.125 mg/mL polymer, 2.5 mg/mL protein, 50 mM phosphate buffer, pH 7.5)

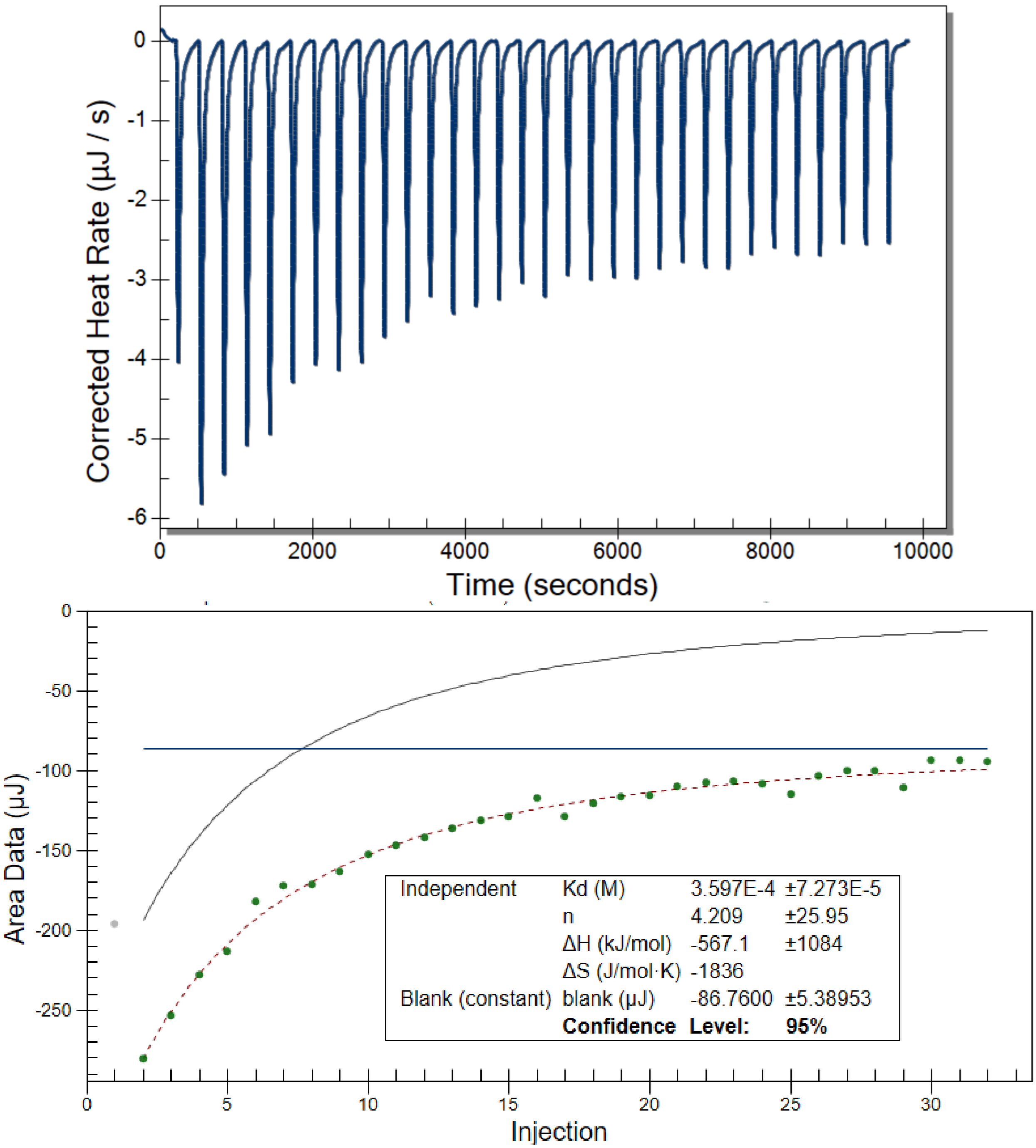

Fig. S7. ITC titration of PCPP with PEG (1 mg/mL PCPP, 20 mg/mL PEG (5 kDa), 50 mM phosphate buffer, pH 7.5)

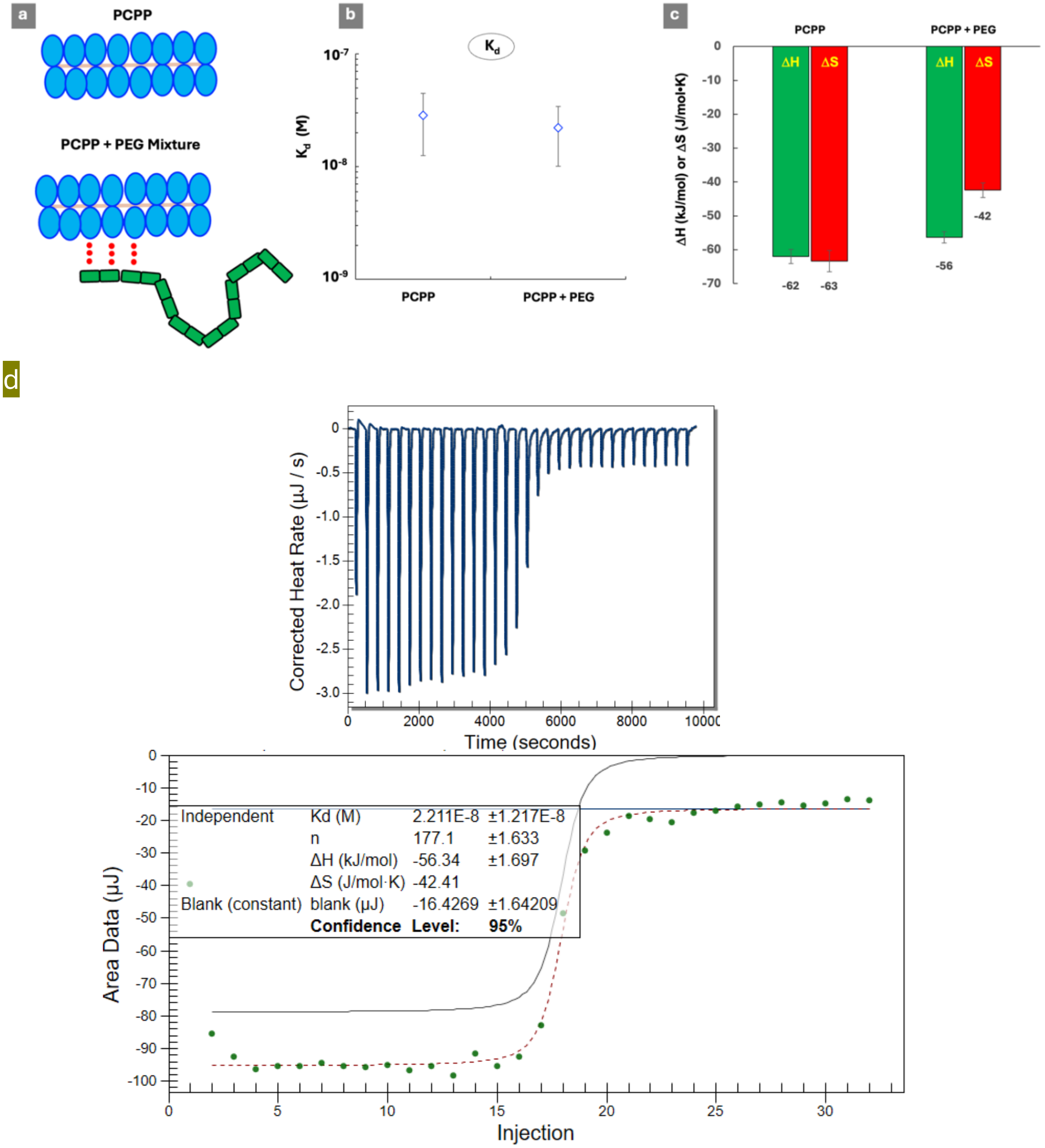

Fig. S8. ITC titration of PCPP and PEG mixture with lysozyme: (a) schematic of PCPP-PEG interactions via formation of hydrogen bonds, comparison of (b) dissociation constants of PCPP – lysozyme and (c) thermodynamic patterns of polymer-protein interactions in the absence and presence of PEG and (d) ITC raw data (0.125 mg/mL polymer, 0.1 mg/mL PEG (5kDa), 2.5 mg/mL protein, 50 mM phosphate buffer, pH 7.5)

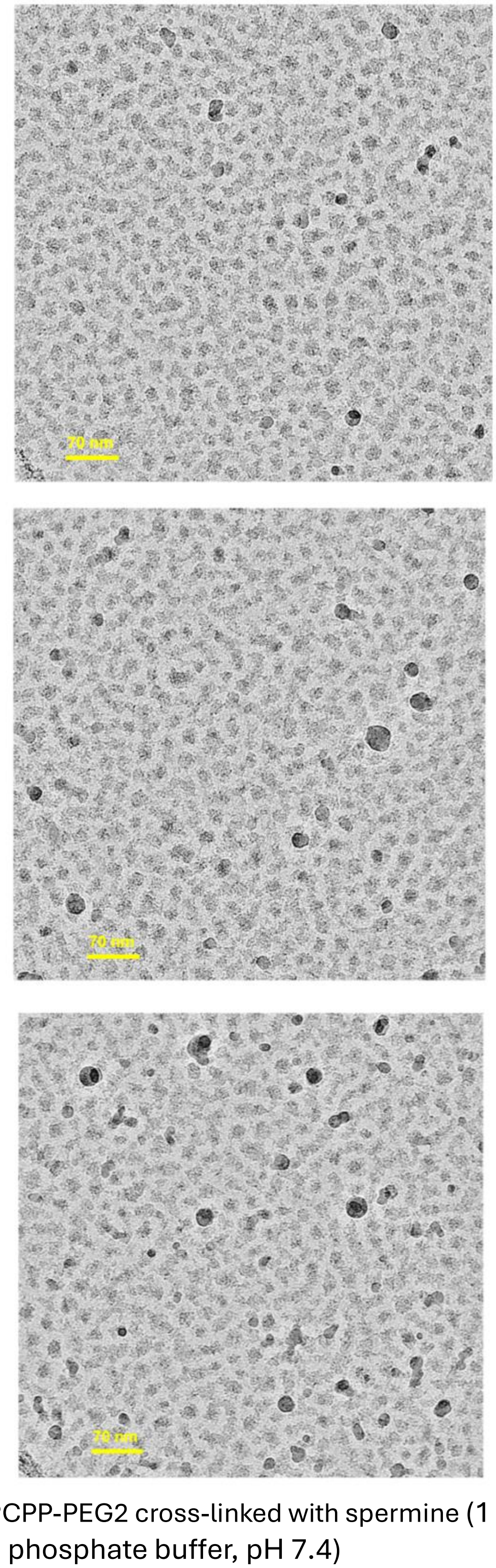

Fig. S9. CryoEM images of PCPP-PEG2 cross-linked with spermine (1 mg/mL PCPP-PEG2, 1mg/mL spermine, 50 mM phosphate buffer, pH 7.4)

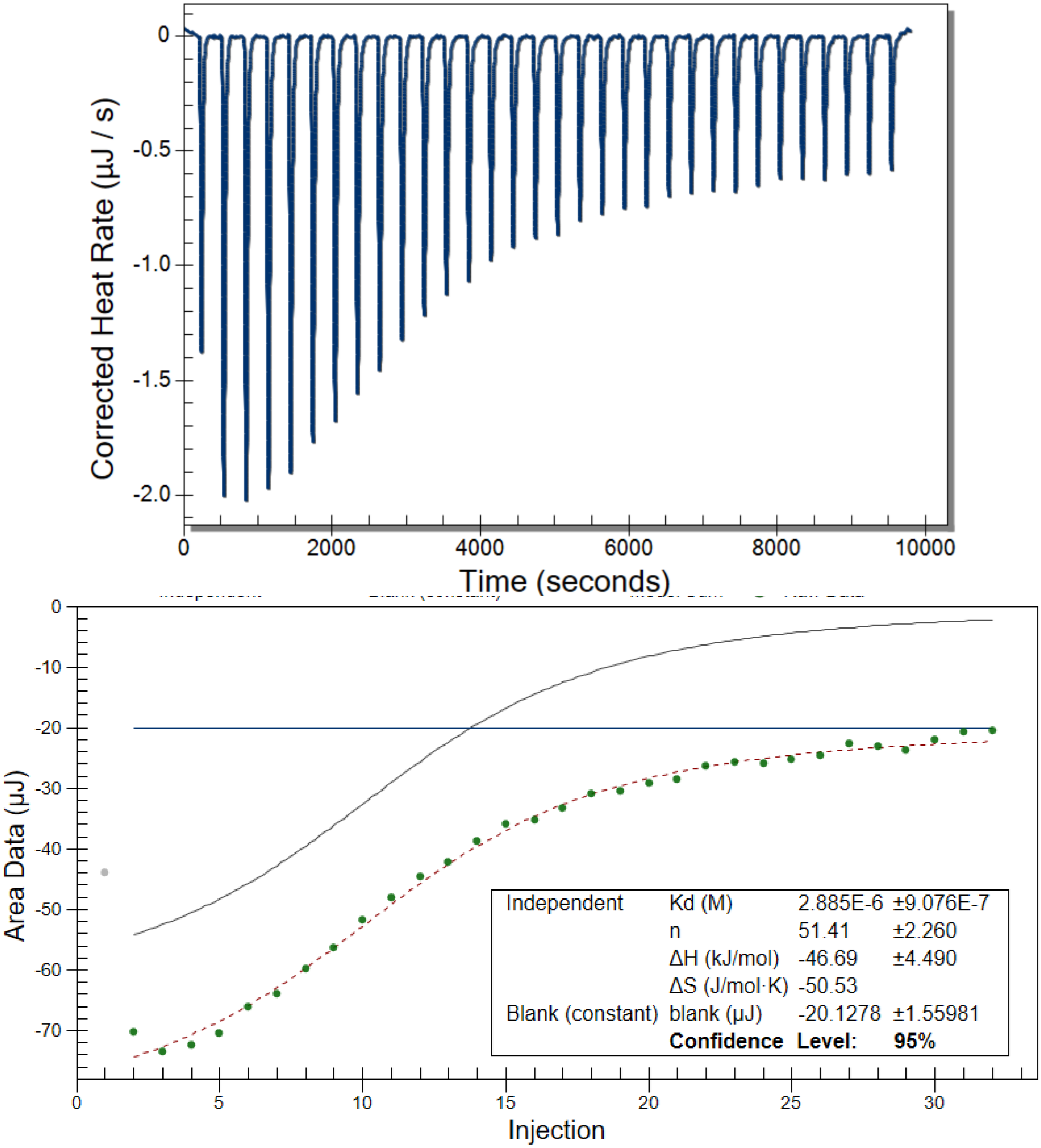

Fig. S10. ITC titration of spermine cross-linked PCPP-PEG2 (NG-01) with lysozyme (0.125 mg/mL nanogel, 2.5 mg/mL protein, 50 mM phosphate buffer, pH 7.5)

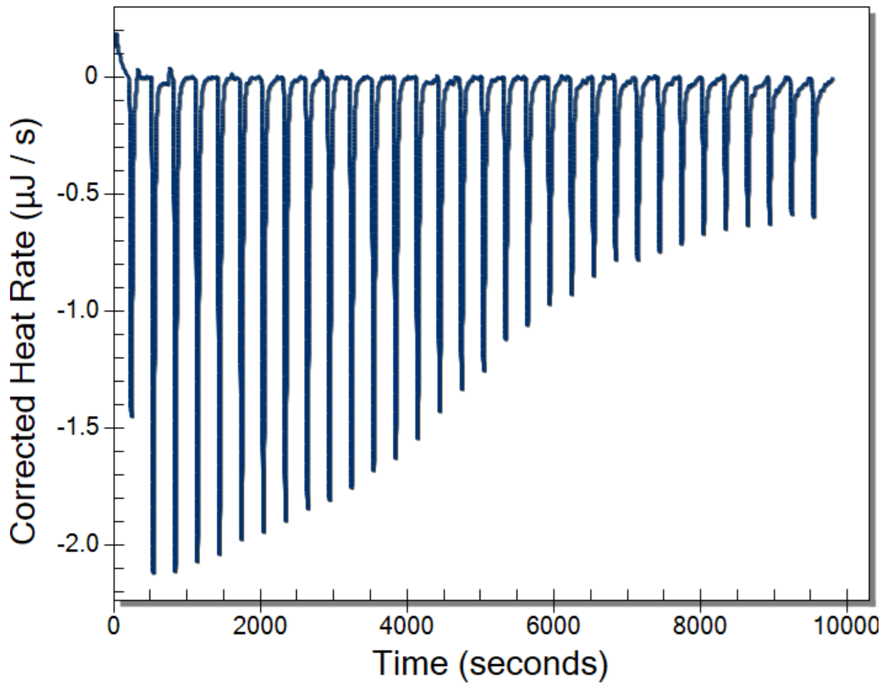

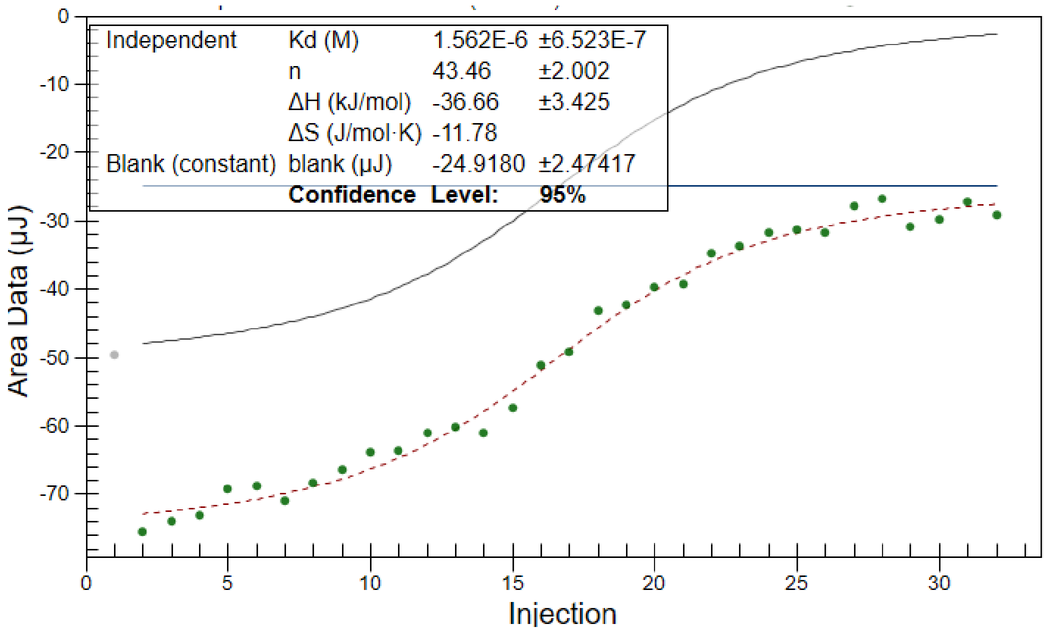

Fig. S11. ITC titration of PCPP-PEG2 with lysozyme in the presence of 50 mM of potassium chloride (0.125 mg/mL polymer, 2.5 mg/mL protein, 50 mM phosphate buffer, pH 7.5)

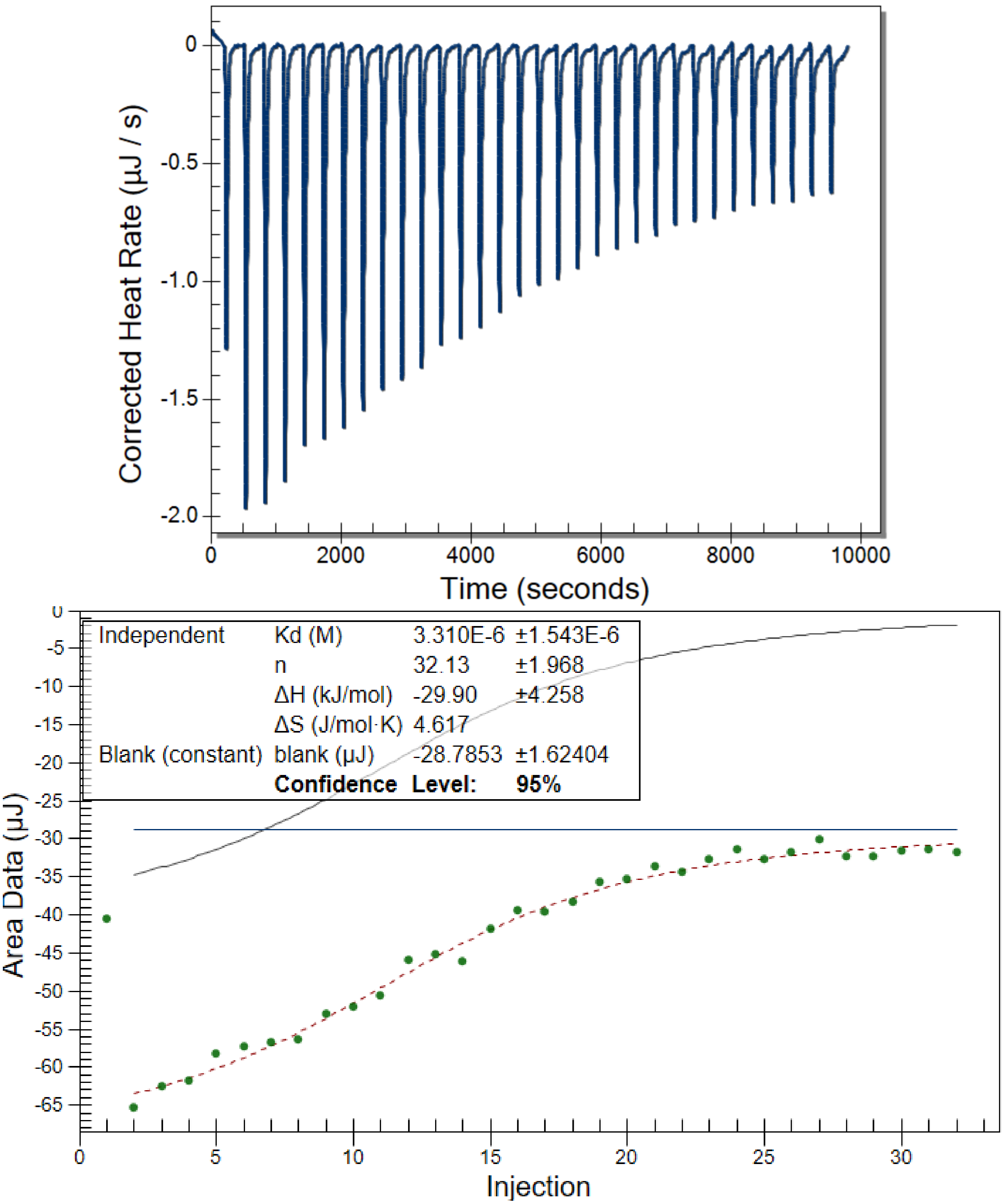

Fig. S12. ITC titration of PCPP-PEG2 with lysozyme in the presence of 100 mM of potassium chloride (0.125 mg/mL polymer, 2.5 mg/mL protein, 50 mM phosphate buffer, pH 7.5)
